# Feature Extraction of Lubricating Oil Debris Signal Based on Segmentation Entropy with an Adaptive Threshold

**DOI:** 10.3390/s24051380

**Published:** 2024-02-21

**Authors:** Baojun Yang, Wei Liu, Sheng Lu, Jiufei Luo

**Affiliations:** 1 College of Mechanical and Vehicle Engineering, Chongqing University, Chongqing 400044, China; 20190701160g@stu.cqu.edu.cn (B.Y.); 202107131321@stu.cqu.edu.cn (W.L.); 2 Chongqing Huashu Robotics Co., Ltd., Chongqing 400714, China; 3 School of Advanced Manufacturing Engineering, Chongqing University of Posts and Telecommunications, Chongqing 400065, China; lusheng@cqupt.edu.cn

**Keywords:** inductive sensors, segmentation entropy, adaptive threshold, noise suppression

## Abstract

Ferromagnetic debris in lubricating oil, serving as an important communication carrier, can effectively reflect the wear condition of mechanical equipment and predict the remaining useful life. In practice application, the detection signals collected by using inductive sensors contain not only debris signals but also noise terms, and weak debris features are prone to be distorted, which makes it a severe challenge to debris signature identification and quantitative estimation. In this paper, a debris signature extraction method established on segmentation entropy with an adaptive threshold was proposed, based on which five identification indicators were investigated to improve detection accuracy. The results of the simulations and oil experiment show that the proposed algorithm can effectively identify wear particles and preserve debris signatures.

## 1. Introduction

Detection of non-stationary short-time pulse signals is encountered in many areas of engineering applications [1]. Pulses might be presented in various forms on account of the physical mechanisms [2,3,4,5]. For example, in the field of lubricating oil monitoring, the signature of induced voltage signals, generated by the inductive debris detection sensors, was similar to a single-period sinusoidal [6]. As a result, detection of the characteristic feature in the form of a sine-like pulse is the main task in signal processing of induced voltages [7,8,9,10]. However, in the actual application, the induced voltages generated by tiny debris passing through the oil channel are extremely weak and would be contaminated by external noise and electric pulses [11,12], thus causing great challenges in target feature identification and extraction.

In recent decades, substantial efforts have been invested in optimizing the structure of inductive sensors and enhancing feature extraction, thus refining the detectability of inductive sensors. In [13], Hong et al. analyzed the axial and radial magnetic field density distributions, clarifying that the uniformity and strength of the axial magnetic field were superior to those of the radial magnetic field under the same excitation condition. Based on this fact, a new inductive sensor with two excitation sources was designed. However, the magnetic field density within the oil tube was still weak, constraining its application in detection of abnormal wear in the early stage. Recently, an inductive debris detection sensor with a symmetrical structure was designed by Feng et al. [14], which could generate a high-gradient magnetic field at the air gap of the magnetic poles. Compared with the radial magnetic field, the high-gradient magnetic field in [14] was much stronger, which made it more conducive to detecting iron particles with a diameter less than 100 μm. Although optimizing the sensor structure greatly promotes the detection performance of the sensors, accurate identification and counting of ferromagnetic particles remain a tough task due to noise, electric pulse, and harmonics. Therefore, once the sensors were ready, signal processing would be one of the most effective and reliable approaches to enhance the performance. So far, many methods for feature extraction have been investigated, which can be generally divided into two categories: the decomposition-based algorithms and non-decomposition-based algorithms. Among them, the decomposition-based methods operate under the assumption that the target signal and background noise can be effectively separated into distinct bands in the transform domain. However, due to the broad energy distribution across the frequency domain, debris signals may be distributed across multiple bands. Consequently, zeroing the coefficients associated with non-target signals inevitably introduces distortions and deformations in the features. This issue becomes particularly pronounced when the frequency band of the target signal overlaps with frequencies of undesired components, significantly diminishing the effectiveness and robustness of these algorithms and potentially resulting in the complete loss of the debris signal.

The non-decomposition algorithms are to employ adaptive filtering to improve the signal-to-noise ratio (SNR). In [15], a detection system with dual inductive units was established by Hong et al., in which a hybrid method based on band-pass filtering and correlation analysis was proposed. This method remarkably improved the ability to detect the induced voltage signals whose central frequency is close to the frequency of interference. Nevertheless, the distortion or deformation of debris signatures were still significant in case that low SNR was encountered. To keep the characteristics intact, a fractional-calculus-based denoising technique was developed in [16], which worked as a low-pass filter. This approach can reduce high-frequency noise while preserving the morphological features. Unfortunately, comparison studies indicated that a large amount of in-band noise would remain in the filtered results, hindering the accurate identification of tiny particles. 

In order to achieve robust noise rejection while maintaining optimal performance in feature extraction, this paper introduces a weighting filtering method based on segmentation entropy. The fundamental working principle of the proposed algorithm involves localizing and segmenting the target signal according to an adaptive threshold. Subsequently, debris identification indicators are applied to eliminate spurious signal blocks, enhancing the statistical accuracy of detection results. Notably, since the operations are based on segmentation entropy with an adaptive threshold and do not rely on any specific mathematical model of debris signals, the proposed algorithm has the advantages of excellent adaptability and compatibility, making it suitable for application in inductive debris sensors with varying types and structures.

## 2. Debris Feature Extraction Algorithm

**Figure 1:**
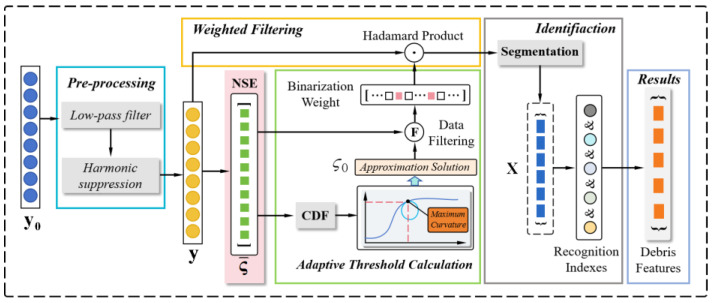
Flowchart of the proposed algorithm.

In this section, a debris feature extraction algorithm based on the normalized segmentation entropy (NSE) with an adaptive threshold is proposed. Initially, during the signal pre-processing step, the harmonics and high-frequency noise are suppressed. Subsequently, the segmentation entropy of pre-processed samples is calculated. The curvature-based threshold is determined through an analysis of the statistical properties of the normalized segmentation entropy. As a result, the samples are divided into discontinuous data blocks, potentially containing target signals, based on the adaptive threshold. To enhance measurement accuracy, five indicators are computed to eliminate spurious debris signals. The main framework of the proposed algorithm is depicted in Figure 1.

### 2.1. Signal Pre-Processed

**Figure 2:**
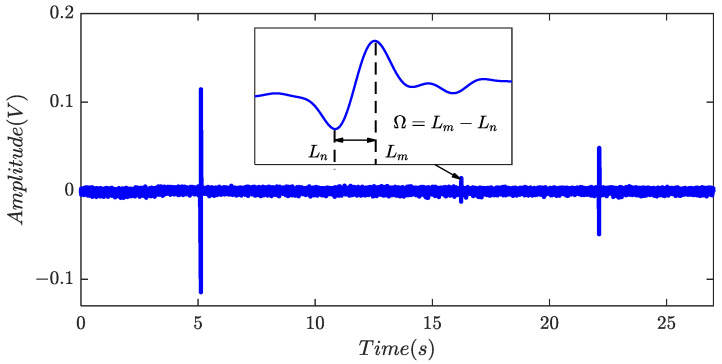
The output signal of debris sensor.

Although debris detection sensors were designed with different structures, the induced voltages generated by debris have a similar shape, which usually approaches to a single-period sinusoidal [6], as illustrated in Figure 2. In addition, the output of sensors would also include random noise, electric pulses, and harmonics. Harmonics are mainly caused by alternating current supply, motion motors, or mechanical vibration. Electric pulses are created by the sudden change of a static magnetic field or unexpected shock vibrations [17]. Several factors account for the random noise in acquired data, including random electromagnetic noise, circuit noise ambient noise and so on. In [17], high-frequency components were suppressed by low-pass filter and harmonics were removed by frequency component canceling with parameter estimation, respectively. To protect the geometric signatures of debris signals, the cut-off frequency of the low-pass filter was suggested to be at least 2.5 times that of the debris central frequency, *f*_d_. In this paper, the frequencies of harmonic components were estimated by the iterative-interpolated discrete Fourier transform algorithm, while the compensation method in the frequency domain was adopted to estimate the amplitudes and phases [18,19].

### 2.2. Normalized Segmentation Entropy Detection

Information entropy, as a physical quantity describing system chaos, can effectively capture the uncertainty of random events [20,21]. Typically, low-frequency random noise can be considered a stationary stochastic process, whereas debris signals and electrical pulses represent abrupt changes. In essence, the presence of debris signals or electric pulses in the samples results in a notable diversity of entropy values. Therefore, the localization of debris signals can be effectively accomplished in the time domain through threshold-based segmentation. 

Denote $y=\{y_{0},y_{1},\ldots ,y_{N-1}\}$ as an arbitrary data block from pre-processed signals with a fixed length, *N*. Inspired by information entropy [20,21], the indicator describing the uncertainty of y can be defined as

(1) $$\varsigma =N\times \log S_{N}$$

where *S_N_* is the variance of y. In this sense, the indicator corresponding to each sample can be calculated according to the sample itself and a certain number of neighbors on each side. In this way, an indicator vector corresponding to a frame of data can be thus gained by $\varsigma =\{\varsigma_{1},\cdot \cdot \cdot \varsigma_{j},\cdot \cdot \cdot \varsigma_{J-1}\}$, where *j* is the number of the siding window. To facilitate the determination of threshold, normalization has been carried out, denoted as

(2) $$\bar{\varsigma }=\frac{\hat{\varsigma }}{{\left\|\hat{\varsigma }\right\|}_{\infty }}$$

In Equation (2), $\hat{\varsigma }$ is the decentralized vector of $\varsigma =\{\varsigma_{1},\cdot \cdot \cdot \varsigma_{j},\cdot \cdot \cdot \varsigma_{J-1}\}$ and ||∙||_∞_ represents the infinite norm of a vector. The elements are normalized in the range from −1 to 1. 

### 2.3. Adaptive Threshold Determination and Segmentation

Once segmentation entropy has been obtained, threshold segmentation can be performed. With a given threshold, time samples with segmentation entropy lower than the threshold are eliminated, automatically dividing the acquired samples into a certain number of small data blocks. The data blocks may contain inductive voltages generated by oil debris. Obviously, determining the threshold is crucial for accurately extracting debris features. If the selected threshold is extremely small, a large number of data blocks may contain merely random noise, significantly increasing the computational burden in subsequent identification operations. On the contrary, an excessively large threshold might lead to severe distortion or even the eradication of target signals.

**Figure 3:**
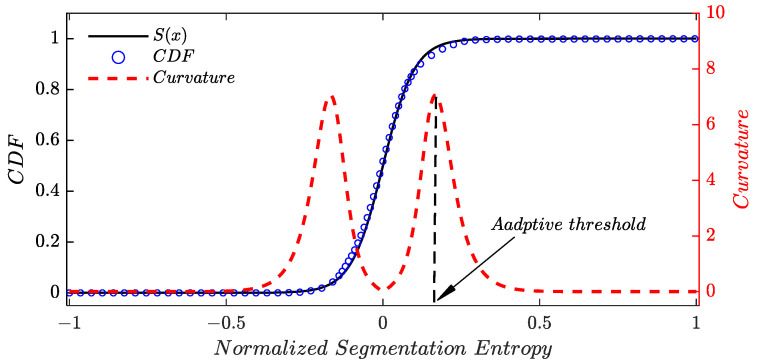
The principle determination of adaptive threshold.

In this paper, the threshold is adaptively determined by the statistical property of analyzed samples. Denote *X* as a random variable representing segmentation entropy, an empirical cumulative distribution of *X* can be obtained according to the elements in $\bar{\varsigma }$. The abscissa value of the point possessing a maximum curvature in cumulative distribution function is selected as the threshold for $\bar{\varsigma }$, as shown in Figure 3. It should be pointed out that computation of discrete curvature is time consuming and the results are strongly dependent on the curvature estimation algorithm. As a result, the Sigmoid function, which has a similar trend with the empirical cumulative distribution function of *X*, was adopted to compute the curvature. Sigmoid function [22] can be expressed as

(3) $$S\left(x\right)=\frac{1}{1+e^{-cx}}$$

where *c* ∈ R^+^ is an adjustable parameter for a good fitting of the empirical cumulative distribution function. Taking the derivative of Equation (3) and substituting *x* = 0, parameter *c* can be simply expressed as *c* = 4*η*, where *η* denotes the discrete slope of the empirical cumulative distribution at *x* = 0. As long as *S*(*x*) is determined, the corresponding curvature can be acquired by

(4) $$\rho \left(x\right)=\frac{\left|S^{\prime \prime }\right|}{{\left(1+S^{\prime 2}\right)}^{3/2}}$$

It is noted that the abscissa value corresponding to the maximum curvature essentially represents the extremum point with respect to the first derivative of curvature; thus, an analytical solution for the segmentation threshold can be derived by locating this extremum. Specifically, if *c* > 6 can be guaranteed, the threshold can be straightforwardly approximated by (see Appendix A)

(5) $$\varsigma_{0}\approx \frac{\ln2c}{c}$$

### 2.4. Identification and Counting of Debris Signal

Following threshold segmentation, the resultant non-zero blocks may contain electric pulses, residual noise or debris signals. Therefore, to further bolster the reliability and accuracy of the debris statistics, the numerical feature extraction and debris identification are imperative. Leveraging the geometric characteristics inherent to debris signals, five identification indicators have been proposed.

(1) ***Index of time sequence.*** Although the magnitudes of debris signals may vary, their waveforms typically exhibit a consistent pattern, wherein the former half is situated below the horizontal axis and the latter half is predominantly positive, as illustrated in Figure 2. Assuming the presence of non-zero blocks covering *L* elements, denoted as ∆ = {∆_0_, ∆_1_, …, ∆*_L_*_−1_}, the time sequence index *β* can be defined as
  (6) $$\beta =\left\{\begin{array}{l} 1,\;\Omega >0\\ 0,\Omega <0 \end{array}\right.$$

In Equation (6), Ω = *L_m_* − *L_n_*, where *L_m_* and *L_n_* represent the sampling indices of the maximum and minimum elements, respectively. A value of *β* = 1 signifies that the fragment adheres to the fundamental criteria of a typical debris signal in terms of its morphological characteristics. Conversely, if *β* = 0, the block can be directly excluded as a non-target signal, thereby immediately halting subsequent operations to reduce the computation.

(2) ***Index of zero point.*** Another distinctive characteristic of debris signals is the presence of a singular zero-crossing point between *L_m_* and *L_n_*. If multiple zero-crossings emerge within this interval, it suggests fluctuations within the acquired samples, a condition inconsistent with the sinusoidal pulse-like nature. Consequently, such occurrences warrant classification of the data block as a non-debris signal. An index in consideration of the zero point can be defined as
  (7) $$\zeta =\left\{\begin{array}{l} 1,\;\upsilon =1\\ 0,\;else \end{array}\right.$$

In Equation (7), *v* denotes the number of the zero-crossing point. Once *ζ* = 0, the block can be excluded, obviating the need for further discrimination due to computational considerations.

(3) ***Index of edge feature***. A high entropy may occasionally occur in noise-only parts that contain outliers; therefore, the corresponding samples would be retained for further processing. However, the segmentation entropy drops rapidly once the outliers are no longer covered by the data window. As a result, residual noises typically have a sharp decrease at the edges of the data block, resulting in a much shorter duration of data edges compared to that of debris signals. Based on the characteristics of debris signals, the data edge, extending from the peaks to the end points, typically persists for a duration of at least Ω samples. Although the weaker edge features may be susceptible to elimination during noise reduction or threshold segmentation processes, the duration of data block edges remains a valuable feature for discriminating against spurious debris signals. An indicator based on the edge feature of the data block can be defined as

(8) $$\xi =Q\left(\frac{L_{n}}{\Omega }\right)\times Q\left(\frac{L-L_{m}}{\Omega }\right)$$

where *Q*(·) represents the function about *ε* defined as

(9) $$Q(\varepsilon )=\left\{\begin{array}{l} 1,\;\varepsilon \geq 1\\ \varepsilon ,\;\varepsilon <1 \end{array}\right.$$

In Equation (8), *ξ =* 1 represents the high possibility that the segmented block possesses satisfactory edge features. On the contrary, if *ξ* << 1, it should be categorized as non-target signals.

(4) ***Index of offset feature.*** Since the induced voltages generated by debris hold a symmetrical pattern, the samples below the zero line and the samples above the zero line would have the similar behavior. Therefore, the sum of maximum and minimum samples in data block would be very small. For residual noise and electric pulses, the bias would be easily observed because they can hardly possess a regularly symmetrical pattern. In order to get rid of the non-debris data, the indicator, describing the offset, is defined as
  (10) $$\gamma =1-|\frac{\max(\Delta )+\min(\Delta )}{\max(\Delta )-\min(\Delta )}|$$

The range of  γ  is [0, 1]. A lower value of  γ  indicates the signal has a higher bias degree. If the indicator approaches to one, the segmented block should be retained.

(5) ***Index of energy feature.*** Although the most of non-target data blocks can be excluded by the former index, there are a few of noise samples that can still meet the requirement. For example, a series of consecutive low frequency noises may contain a sub-sequence, the pattern of which is very similar to a sine-like signal. In this case, the noise samples might be wrongly classified as a debris signal. To reduce the false discrimination rate, an index related to the energy feature is proposed

*η* = *E_d_*/*E*_0_ (11)

where *E_d_* and *E*_0_ are the energy of main-body of target signal and the energy of all acquired data samples, respectively. It is evident that 0 ≤* η* ≤ 1. A small value of *η* indicates a higher likelihood of noise patterns, as it suggests that the energy is dispersed across a wide range rather than concentrated within the main body. Indeed, this phenomenon differs significantly from the pattern of debris signals.

After calculating the five indicators, potential target signal fragments can be discriminated. It should be noted that a lower threshold value for the last three indicators is typically chosen to account for noise and interference. However, this may result in the inclusion of non-target blocks in some cases. To refine the statistics, a global threshold (GT) for the product of the five indicators is required. In the subsequent sections of this paper, the thresholds for each indicator are set as follows: *β* = 1, *ζ* = 1, *ξ* > 0.7, *γ* > 0.7, *η* > 0.7 and GT > 0.6. The main steps of the segmentation entropy-based feature extraction algorithm are summarized in Table 1.

## 3. Simulation and Results

### 3.1. Verification of the Adaptive Threshold

**Figure 4:**
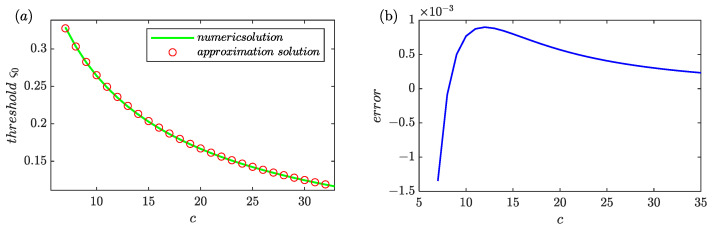
The thresholds as a function of *c* computed by numeric method and the approximation solution errors. (**a**) The solutions computed by numeric method and the analytic solution computed by Equation (5). (**b**) The errors between the exact solution and the approximation solution.

First, in order to examine the errors between the exact solution and the analytic approximation solution of Equation (5), the adaptive thresholds as an increase of *c* were computed and the results were shown in Figure 4a. It can be observed that the two solutions had the same trend and coincided well with each other. The errors were shown Figure 4b and all values were kept at a small stage for *c* > 7. If *c* > 8 can be guaranteed, the errors could be almost ignored because the absolute difference was reduced to a level less than 0.001. As a result, the adaptive threshold can be easily determined through the straightforward approximation solution so long as the parameter *c* has been obtained.

**Figure 5:**
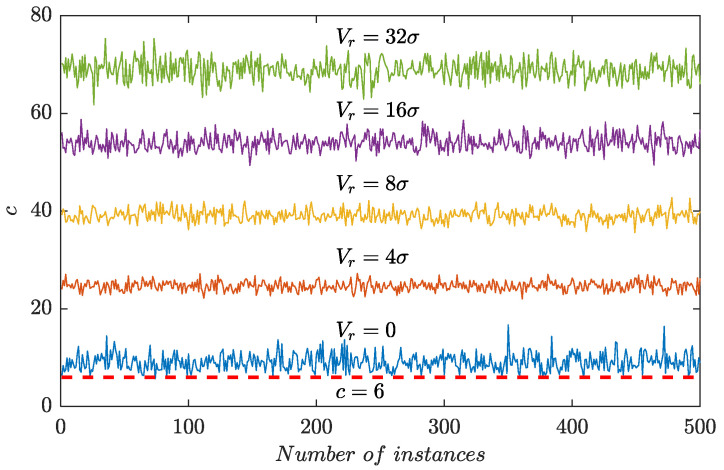
*c* for 500 independent trails.

Figure 5 displayed the values of *c* for 500 independently sampled segments of contained Gaussian random noise with a zero mean and a variance σ^2^ = 1. Each segment comprised 1,000,000 discrete samples, and a short sine-like wave was randomly added to simulate the induced voltages generated by wear debris. In Figure 5, *V_r_* represented the amplitude of the single-period sine wave. In the absence of debris signals (*V_r_* = 0), the values of *c* hovered around 10 but never reached the baseline (*c* = 6). When debris signals were introduced, the values of *c* increased and they hovered over 20 for *V_r_* = 4σ. As *V_r_* went up, the average value of *c* continuously climbed and reached 70 for *V_r_* = 32σ. The simulation results indicated that *c* > 8 can be satisfied in a wide range of possible cases and the adaptive threshold computed by Equation (5) could reasonably hold.

### 3.2. Verification of the Feature Extraction and Identification

**Figure 6:**
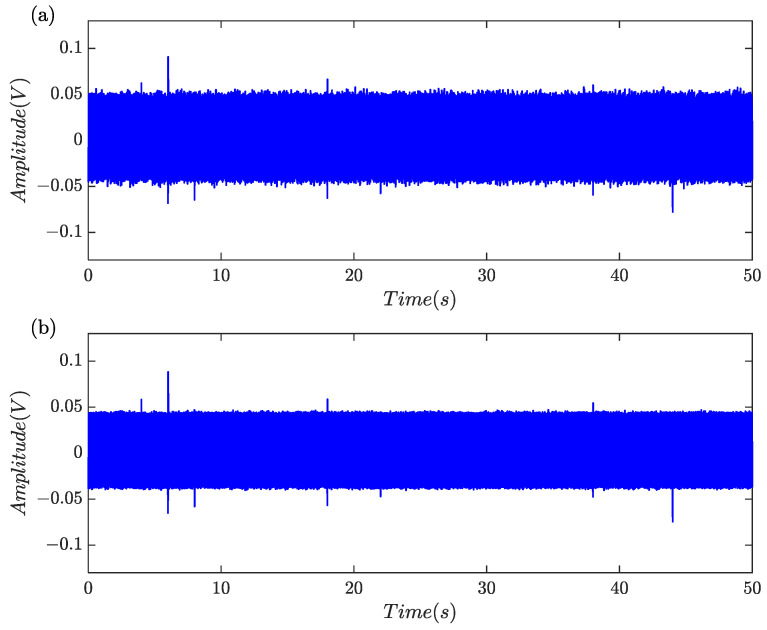

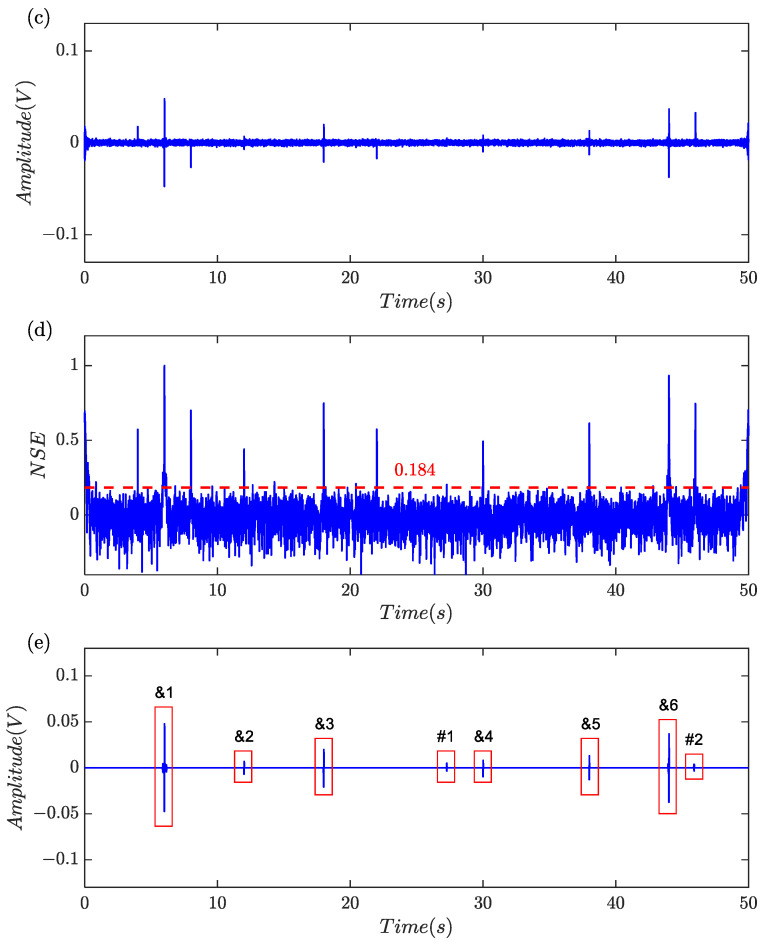
Feature extraction by the proposed algorithm. (**a**) Simulated signal; (**b**) signal after low-pass filtering; (**c**) signal after harmonics rejection; (**d**) the normalized segmentation entropy and adaptive threshold; (**e**) segmentation and identification results.

A numeric experiment was carried out to examine the effectiveness of the proposed algorithm outlined in Table 1. Six separate single periodic sinusoidal signals with different amplitude were created to model the debris-related induced voltages. The amplitude of the induced voltages was set to 0.050, 0.006, 0.020, 0.008, 0.012, 0.040, respectively. Harmonics, triangular pulses and random noise were also simultaneously included to demonstrate the practicality of the approach. Sampling rate is 5 kHz and a total of 250,000 samples equal to a time frame of 50 s were generated. The simulation signal was illustrated in Figure 6a and it was shown that harmonics substantially masked the features of induced voltages since it was difficult to find the characteristics of a debris-related signal induced by the tiny ferromagnetic particles.

Figure 6b showed that the samples after low-pass filtering and harmonics remained significant. Figure 6c showed that the samples after harmonics rejection and the induced voltages with large amplitude can be clearly observed. The normalized segmentation entropy was shown in Figure 6d and the threshold was about 0.184. A total of 36 data blocks were obtained through threshold segmentation, of which eight were retained via feature identification. For clarity in observation, the eight data blocks were labeled and categorized into two groups, as shown in Figure 6e. Considering that the global indicators for #1 and #2 were 0.5393 and 0.4508, respectively, they were classified as non-debris signals. Finally, six simulated characteristic signal blocks were all captured.

**Figure 7:**
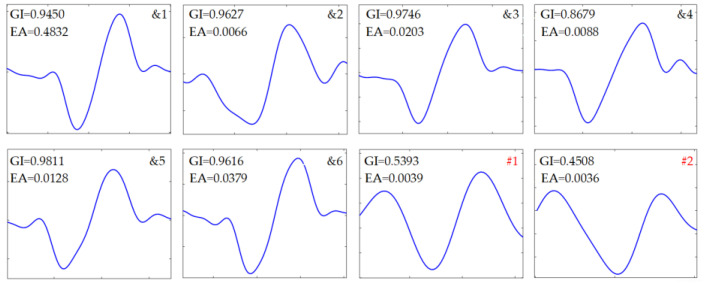
Identification results by the feature indicators and estimated amplitude (EA).

The detailed results obtained by the proposed method were depicted in Figure 7 and the identification indicators of those data blocks were given in Table 2. Six debris-related blocks were identified. The global indicators and the corresponding amplitudes returned by the extracted features were also shown for comparison. It can be seen in Figure 6e that the positions as well as amplitudes coincided well with the pre-settings. It is demonstrated that the debris-related features can be correctly located by computing the normalized segmentation entropy, and non-debris signals can be effectively excluded using the five identification indicators and the global threshold. 

### 3.3. Verification of Robustness of the Proposed Algorithm

To illustrate the robustness of proposed algorithm, 1000 numeric experiments were individually performed. The minimum value of the computed *c* was 19.8, implying that the approximation tactic was tenable. Discrimination rates (DR) of debris features were shown in Table 3, and it was reflected that all six virtual particles can be identified in most cases. The mean value (MV) and standard deviation (STD) of the extracted amplitudes were also given in Table 3. The mean values were very close to the pre-settings and the standard deviations were on the level of 5.0 × 10^−^^4^~9.0 × 10^−^^4^, indicating the effectiveness and robustness of the method. Table 4 shown a few of the residual data blocks, which were unable to be excluded by the indicators and, as a result, were classified as the debris-induced segments. Apparently, false discrimination was a small-probability event and the amplitudes of spurious debris signals were all very minute. Such occasional cases can hardly influence the statistics of wear debris, therefore almost being ignored in practical equipment monitoring. 

From the above experiments, it can be inferred that the selection of an appropriate threshold plays a crucial role in signal segmentation and feature identification. A high threshold may obscure the edge features of debris signals, leading to misclassification or even complete removal of target signals. Conversely, a small value will result in a large amount of residual noise, increasing computational load and potentially introducing additional non-target elements into the results, thereby altering detection outcomes. In the experiments, the computed threshold values exhibited slight fluctuations due to variations in noise but generally remained stable, with a maximum of approximately 0.1858 and a minimum of about 0.1736. The optimal threshold value should be around 0.18, as indicated in Figure 5d. Thus, the adaptive threshold proposed in this paper not only demonstrates strong robustness but also possesses accurate discriminability.

## 4. Experiments and Results

### 4.1. Experimental Settings and Data Acquisition

**Figure 8:**
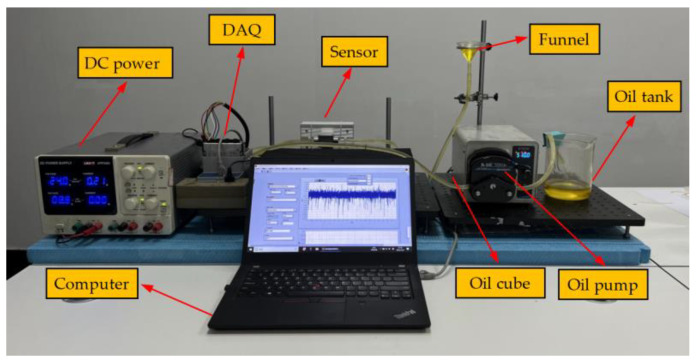
The main structure of experimental platform.

An experimental platform was established to examine the performance of the proposed algorithm in practical application, as shown in Figure 8. In the experiment, seven spherical ferrous particles of different sizes were selected for testing, and the micro-graph of these particles indicated the diameters were approximately 210 μm, 60 μm, 135 μm, 50 μm, 70 μm, 65 μm, 145 μm, respectively. The particles were successively driven across an inductive sensor along with the lubricant. The flow rate was 510 mL/min, indicating about 1.8 m/s of running speed in flow channel. The amplification factor was 2000, the sampling rate was set to 5 kHz, and 250,000 samples were collected, equivalent to a sampling duration of 50 s.

### 4.2. Experimental Results

**Figure 9:**
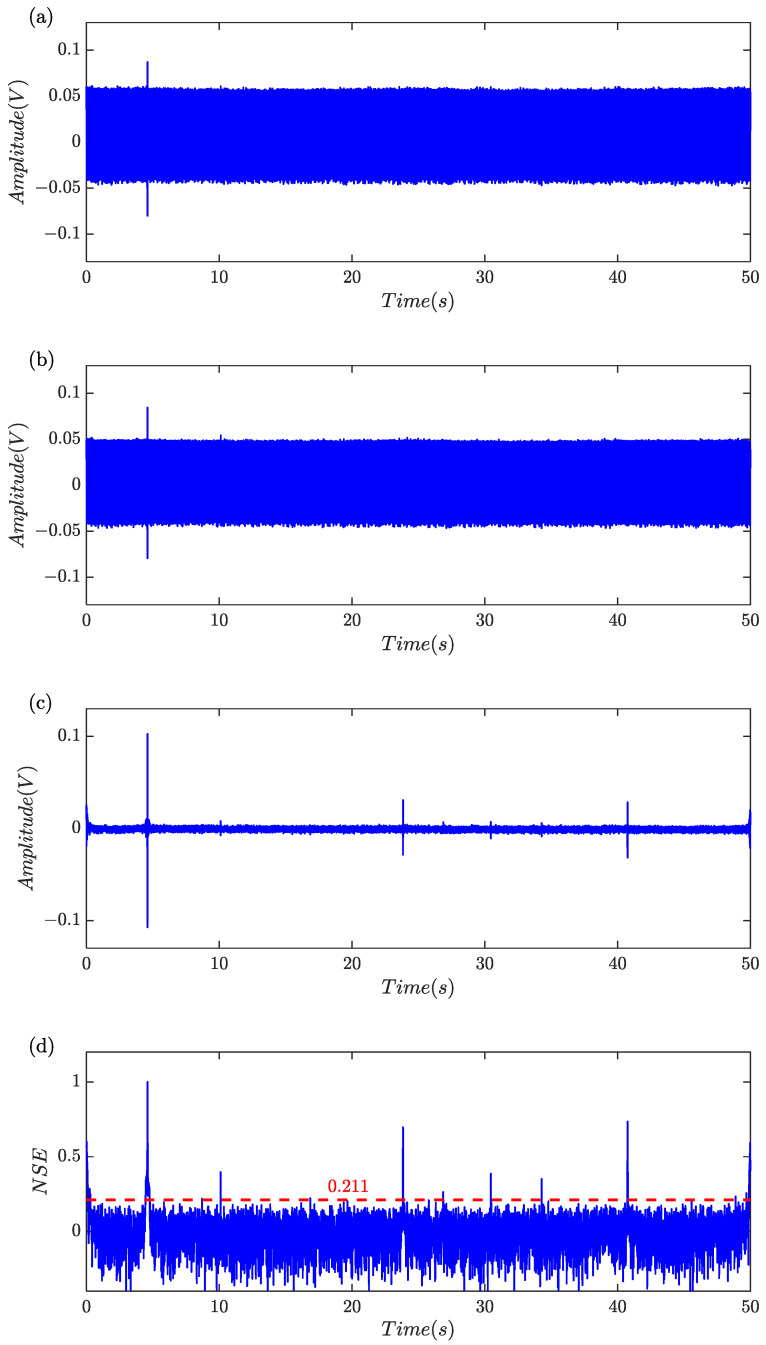

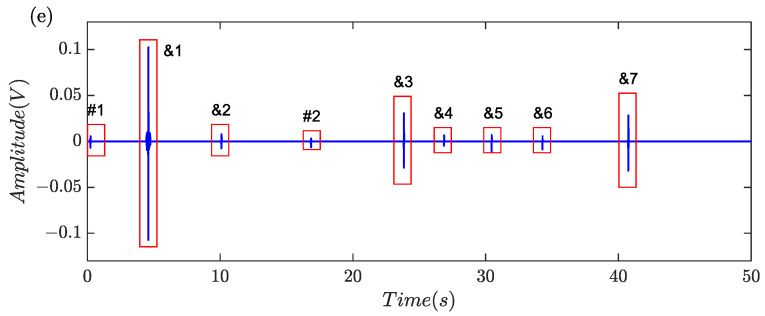
Signal processing results. (**a**) Sampled signal; (**b**) signal after low-pass filtering; (**c**) signal after harmonics rejection; (**d**) the normalized segmentation entropy and adaptive threshold; (**e**) segmentation and identification results.

The collected experimental data is shown in Figure 9a. Due to strong harmonics and background noises, the weak debris signatures could not be discerned. As indicated in Figure 9c, harmonics and high-frequency components were greatly suppressed after pre-processing and SNR was remarkably improved. The signatures generated by the particles with larger volume are clearly visible. However, the weak debris signals were still subjected to the corruption and masking caused by random noises. Figure 9d illustrated the segmentation entropy, where transient jump signals, including debris signatures and electrical pulses, had substantially higher normalized segmentation entropy than those containing merely noises. According to the adaptive threshold determination strategy, *c* = 16.59 is gained and the threshold was about 0.211.

**Figure 10:**
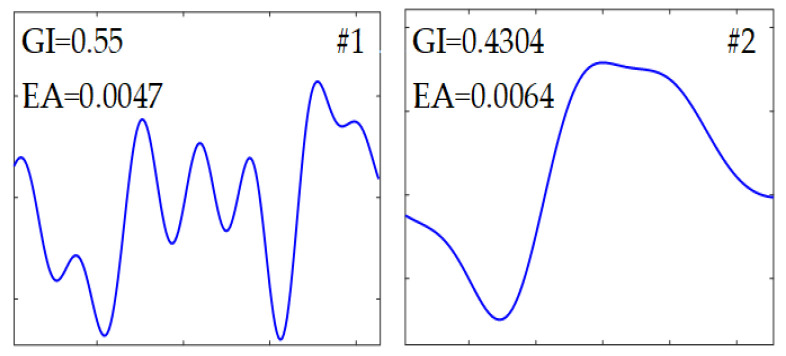
Residual noise blocks.

**Figure 11:**
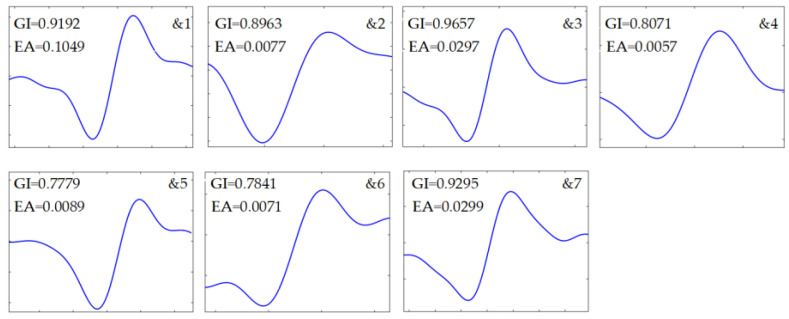
Captured debris signals.

Identification was subsequently performed, and the numerical features are listed in Table 5. Based on the proposed indicators, a total of nine non-zero blocks, labeled as &1 to &7 and #1 to #2, respectively, were retained, as shown in Figure 9e, because all five indicators for each individual were greater than 0.70. However, the product of the indicators is about 0.5508 for #1 and 0.4304 for #2. As a result, the two segments were classified as residual noise, as described in Figure 10, implying that there was no case of false discrimination. The final identification results are shown in Figure 11, where all captured debris signals have a similar shape, resembling a single-period sinusoidal.

### 4.3. Comparison and Discussion

**Figure 12:**
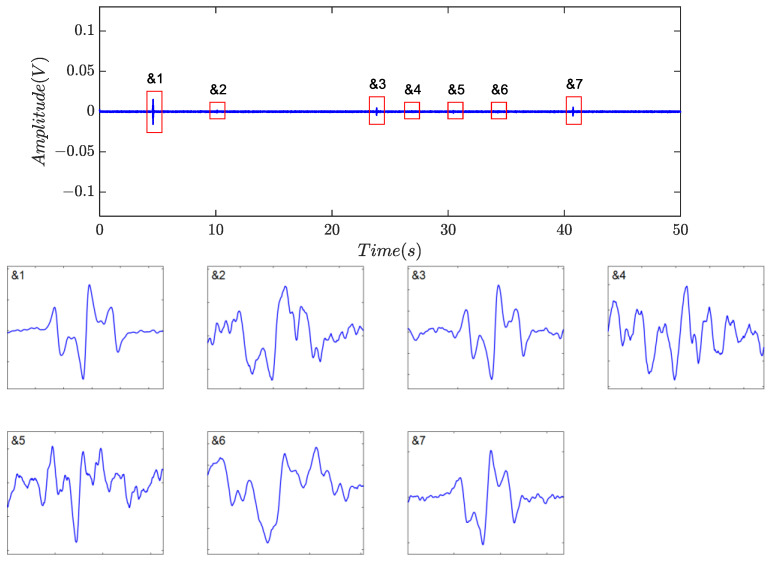
Filtering results of the symplectic geometry mode decomposition in [23].

The denoising algorithms proposed in [16,23,24] were examined for a comparison study. Figure 12 depicted the results obtained using symplectic geometry mode decomposition (SGMD). The collected detection signal, as shown in Figure 9a, underwent decomposition into 119 sub-components. Among these, sub-components 16 to 25 were utilized for reconstructing the debris signals. The results indicated that SGMD effectively suppressed harmonics and random noise; however, noticeable amplitude attenuation of induced voltages was observed. The severe distortion in signal geometrical morphology may pose challenges for feature identification.

**Figure 13:**
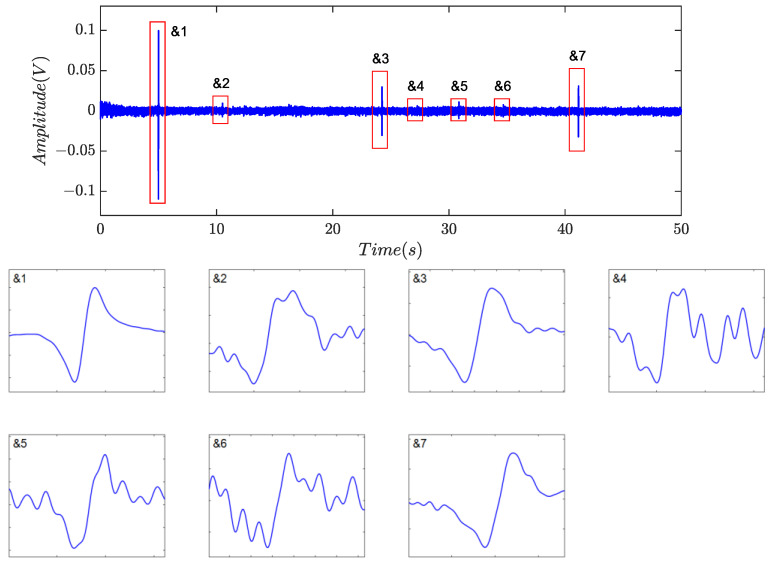
Filtering results of the fractional calculus in [16].

Figure 13 presented the outcomes obtained utilizing fractional calculus with a derivative order of *l_i_* = −0.9. Seven candidates were identified, and the majority of morphological characteristics were preserved, albeit with a slight loss in crest values. Nonetheless, residual background noise remained significant, and further experiments demonstrated that altering the derivative order yielded little improvement in noise reduction. These findings implied that automatic debris feature identification following fractional calculus operation would be of great challenges in practical applications due to the substantial amount of embedded noise. Furthermore, the residual noise poses a hindrance to the detection of small particles, particularly under low signal-to-noise ratio conditions.

**Figure 14:**
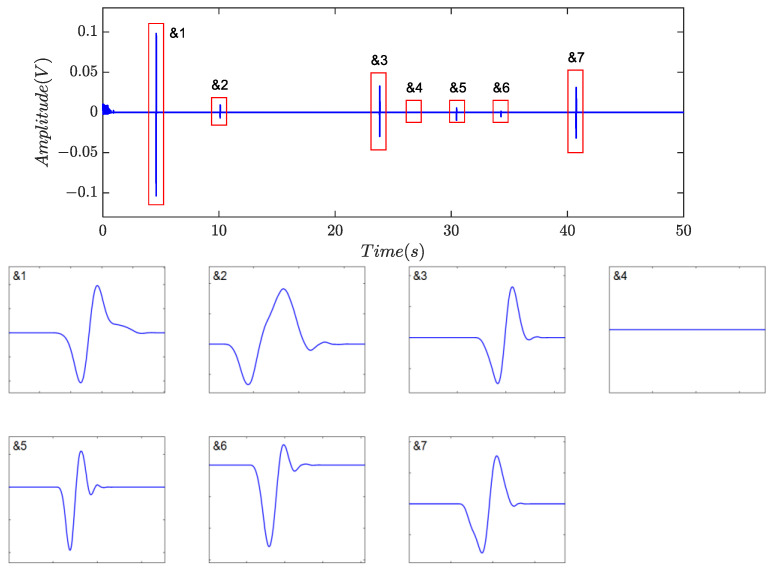
Filtering results of TIWT with *d_l_* = 4 in [24].

**Figure 15:**
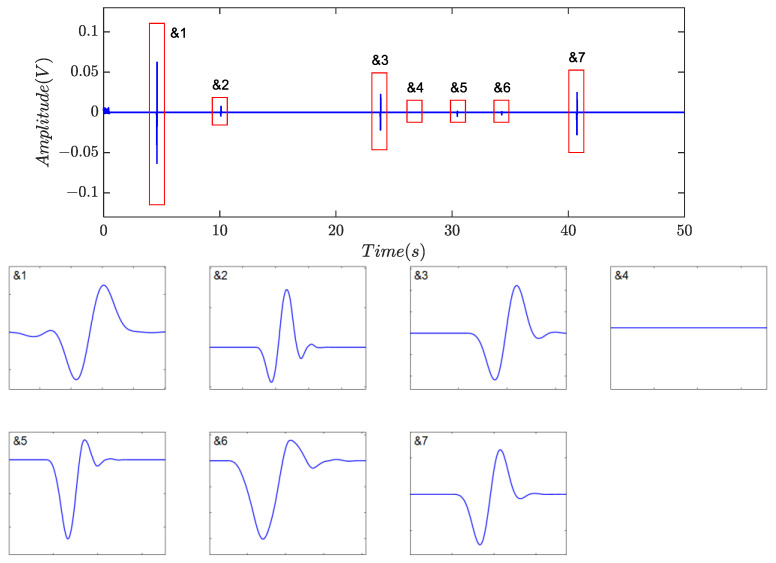
Filtering results of TIWT with *d_l_* = 5 in [24].

**Figure 16:**
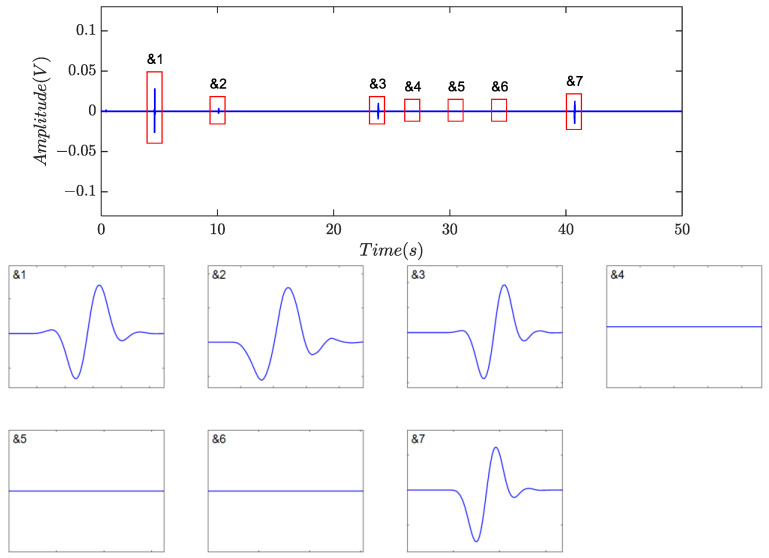
Filtering results of TIWT with *d_l_* = 6 in [24].

Figure 14, Figure 15 and Figure 16 depicted the filtering results using the method based on the time- invariant wavelet transform (TIWT) and kurtosis index, where *d_l_* represents the wavelet decomposition level (WDL). Compared to symplectic geometry mode decomposition and fractional calculus, TIWT exhibited superior performance in harmonic and noise rejection, where four or six possible debris can be left, depending on the choice of decomposition level. As illustrated in the magnified view, waveform distortion and feature eradication were inevitable, particularly with fine particles. In practical scenarios, achieving proper determination of the decomposition level proves challenging, potentially resulting in the loss of target signals. Additionally, the operation of TIWT may introduce endpoint effects, leading to a significant number of fragmented signal segments and thereby affecting the accuracy of statistics.

Overall, even though various strategies have been devised to enhance debris detection performance, the inherent conflict between noise reduction and debris feature preservation remains a difficulty for inductive debris sensors. This paper addresses this challenge by introducing a straightforward and practical signal-processing method, aiming to bridge the gap and resolve the contradiction. The proposed technique demonstrates successful improvements in both noise reduction and feature retention through the introduction of an adaptive threshold, which takes into account the diverse nature of debris signals and undesired components. 

For the pre-processed signals with a sample length of *R*, the computation burden of the proposed algorithm primarily lies in calculating the normalized segmentation entropy $\bar{\varsigma }$. This involves computing the *R*th variance and performing *R*th logarithmic operations, as outlined in Equation (1), along with normalization operations requiring *R* divisions in Equation (2). *η* can be obtained through a single traversal of $\bar{\varsigma }$, and, subsequently, the adaptive threshold can be determined according to Equation (5). Segmentation entails *R* multiplications, while the identification of each short data block involves only a few conventional algebraic operations, resulting in a computational load significantly lower than the preceding procedures. As a result, the proposed algorithm may be possibly deployed for practical application in real-time monitoring systems. The experiments illustrated that the algorithm can reliably detect ferromagnetic particles of 40 μm and above. Importantly, the effectiveness and robustness of this method are achieved without reliance on specific debris signal models. Consequently, this theoretical framework allows for promising application across different inductive debris detection sensors and has the potential for expansion into other pulse-detection-based domains.

## 5. Conclusions

In this paper, a feature-extraction method for debris signals based on information entropy with an adaptive threshold was proposed. Additionally, five identification indicators were designed to eliminate spurious debris signals and enhance detection accuracy. The efficacy and performance of the proposed framework are validated through simulations and an oil experiment. We also analyze the de-noising results of three traditional signal processing methods for comparison. The proposed approach excels in debris identification, feature preservation, and exhibits significant potential for application in other pulse-signal-based detection areas.

## Figures and Tables

**Table 1:** Main steps of the proposed algorithm.

Order	Steps and Algorithms
1	Induced voltages acquisition and data conversation
2	Elimination of high frequency noise using low-pass filtering and harmonics suppression using parameter estimation
3	Calculation of the normalized segmentation entropy $\bar{\varsigma }$ using Equation (2)
4	Determination of the adaptive threshold $\varsigma_{0}$ using Equation (5) and segmentation of the suspected characteristic signal with the adaptive threshold
5	Calculation of the five indicators as well as the global threshold and performing the debris signal identification with β = 1, ζ = 1, ξ > 0.7, γ > 0.7, η > 0.7 and GT > 0.6
6	Amplitude classification and counting

**Table 2:** Identification indicators of the extracted signals.

Order	*ξ*	*γ*	*η*	*GI*
&1	0.9963	1.0000	0.9485	0.9450
&2	0.9971	1.0000	0.9656	0.9627
&3	0.9746	1.0000	1.0000	0.9746
&4	0.9102	0.9643	0.9889	0.8679
&5	0.9919	1.0000	0.9869	0.9811
&6	0.9911	1.0000	0.9702	0.9616
#1	0.8208	0.8333	0.7885	0.5393
#2	0.9451	0.7931	0.6014	0.4508

**Table 3:** The mean value and standard deviation of debris signals.

Order	DR	MV	STD (×10^−4^)
&1	100%	0.0475	8.763
&2	82%	0.0059	6.524
&3	100%	0.0189	5.668
&4	91%	0.0078	7.710
&5	99%	0.0116	5.521
&6	100%	0.0379	8.399

**Table 4:** Identification indicators of noise data blocks.

Order	*ξ*	*γ*	*η*	*GI*	*EA*
I	0.9473	1.0000	0.7786	0.7375	0.0043
II	0.8202	0.7917	0.8806	0.5718	0.0033
III	0.9658	0.8889	0.6878	0.5904	0.0038
IV	0.9048	1.0000	0.8639	0.7817	0.0031
V	0.8183	1.0000	0.9931	0.8126	0.0041
VI	0.8678	1.0000	0.9823	0.8524	0.0038
VII	0.8079	1.0000	0.8173	0.6604	0.0035
X	0.8132	0.7895	0.8565	0.5499	0.0040
IX	0.9485	1.0000	0.7929	0.7520	0.0040

**Table 5:** Identification indicators of debris signals.

Order	*ξ*	*γ*	*η*	*GI*	*EA*
&1	0.9776	1.0000	0.9403	0.9192	0.1049
&2	0.9859	0.9091	1.0000	0.8963	0.0077
&3	0.9657	1.0000	1.0000	0.9657	0.0297
&4	0.8393	0.9615	1.0000	0.8071	0.0056
&5	0.7779	1.0000	1.0000	0.7779	0.0089
&6	0.7887	1.0000	0.9941	0.7841	0.0071
&7	0.9395	1.0000	0.9893	0.9295	0.0299

## Data Availability

The datasets generated during and/or analyzed during the current study are not publicly available due to copyright issues but are available from the corresponding author on reasonable request.

## Appendix A

Based on the content of Section 2, the abscissa value of the point possessing a maximum curvature in fitting curve *S*(x) can be selected as the segmentation threshold. Noticing that the abscissa value of the maximum curvature is essentially the extremum point in corresponding to the first derivative of curvature, an analytical solution as an segmentation threshold can thus be obtained by solving the extremum. According to the Figure 2, the valid segmentation threshold commonly locates in the interval [0, 1]. With regard to a known parameter *c* = 4*k*_0_ (*k*_0_ > 1), the curvature can be denoted as

(A1) $$\rho (x)=\frac{c^{2}t{\left(t+1\right)}^{3}\left(t-1\right)}{{\left[{\left(t+1\right)}^{4}+c^{2}t^{2}\right]}^{\frac{3}{2}}}$$

where $t=e^{cx}(x>0)$. Taking the first derivative, yields

(A2) $$\rho^{\prime }(x)=\frac{-c^{3}t{\left(t+1\right)}^{2}f\left(t\right)}{{\left({\left(t+1\right)}^{4}+c^{2}t^{2}\right)}^{\frac{5}{2}}}$$

in which,

(A3) $$f(t)=t^{6}-2c^{2}t^{4}+2c^{2}t^{3}-2c^{2}t^{2}-9t^{4}-16t^{3}-9t^{2}+1$$

Apparently, the numbers of root for the Equation (A2) depend on the f(t). According to Appendix B, one can conclude that f(t)=0 contains four unequal real roots, among which possess the relationship $\zeta_{1}<\zeta_{2}<0<\zeta_{3}<1<\zeta_{4}$. Therefore, t>1, $\zeta_{4}$ must be the extreme point corresponding to the maximum curvature. However, computation of $\zeta_{4}$ would be difficult. An approximation value with high accuracy can be obtained by solving $t^{6}-2c^{2}t^{4}=0$ as long as the coefficient *c* is large enough. Thus, the analytic solution of the segmentation threshold can be approximately denoted by

(A4) $$\zeta_{4}\approx \sqrt{2}c$$

In fact, the segmentation threshold can be determined from a floating candidate threshold interval, not necessarily at the exact extreme point. A value in the neighborhood can also meet the segmentation requirement, making the approximate solution applicable in practical circumstances.

## Appendix B

The roots for f(t)=0(t∈R) with the qualification of known parameter c∈R can be conducted by seeking a derivative. Taking the first derivative of f(t) yields

(A5) $$f^{\prime }(t)=h(t)t$$

where

(A6) $$h\left(t\right)=6t^{4}-4\left(2c^{2}+9\right)\;t^{2}+6\left(c^{2}-8\right)\;t-2\left(2c^{2}+9\right)$$

It is obvious that $f^{\prime }(t)=0$ contains the root *t* = 0, and the rest are controlled by h(t) Taking the first derivative of Equation (A6), one can obtain

(A7) $$h^{\prime }\left(t\right)=24t^{3}-8\left(2c^{2}+9\right)\;t+6\left(c^{2}-8\right)$$

Given that $h^{\prime \prime }\left(t\right)=0$ has a couple of real roots, $h^{\prime }(-1)>0$ and $h^{\prime }(1)<0$, $h^{\prime }\left(t\right)=0$ must consist of three unequal real roots, which can be denoted as $\xi_{1}<-1<\xi_{2}<1<\xi_{3}$. Note that we can achieve

(A8) $$c^{2}=\frac{3(t-3){\left(t+1\right)}^{3}}{4t^{2}-3t+2}$$

By setting Equation (A6) to zero. For ∀t∈(−1,1), $c^{2}<0$ holds. It is indicated that $h\left(t\right)=0$ has only two real roots and there are no roots for the equation within the interval (−1, 1). Further, it is easy to know $f^{\prime }(t)=0$ exists three unequal real roots, among which can be marked as $\varsigma_{1}<-1$, $\varsigma_{2}=0$ and $\varsigma_{3}>1$. Immediately, one can obtain that $f^{\prime }\left(t\right)<0$ for $t\in \left(-\infty ,\varsigma_{1}\right)\cup \left(\varsigma_{2},\varsigma_{3}\right)$ and $f^{\prime }(t)>0$ for $t\in \left(\varsigma_{1},\varsigma_{2}\right)\cup \left(\varsigma_{3},+\infty \right)$. Combined with f(0)=1 and f(1)<0, we can finally conclude that f(t)=0 contains four unequal real roots $\zeta_{1}<\zeta_{2}<0<\zeta_{3}<1<\zeta_{4}$.
